# Scissor Position of the Orthopedic Table for the Resolution of Intertrochanteric and Subtrochanteric Hip Fractures

**DOI:** 10.7759/cureus.16442

**Published:** 2021-07-17

**Authors:** Cristian Barrientos, Julian Brañes, Rodrigo Wulf, Maximiliano Barahona, Sebastián Carvajal Fuentes

**Affiliations:** 1 Orthopedics and Traumatology, University of Chile Clinical Hospital, Santiago, CHL; 2 Orthopedics, University of Chile Clinical Hospital, Santiago, CHL; 3 Department of Orthopedic Surgery, University of Chile, Santiago, CHL; 4 Orthopedics, Clínica Bupa Santiago, Santiago, CHL

**Keywords:** intertrochanteric hip fracture, subtrochanteric hip fracture, orthopedic table, well leg, scissor position

## Abstract

During the surgical resolution of intertrochanteric and subtrochanteric hip fractures on an orthopedic table, a fluoroscope needs to be used in orthogonal planes. This requires that the contralateral leg does not obstruct the radioscopic view, so patients are often placed in a hemilithotomy position. This position, also called the Lloyd-Davis position, involves hip flexion, hip abduction, hip external rotation, and knee flexion. However, rare complications, such as acute leg posterior compartment syndrome, have been described.

In addition, patients with severe osteoarthritis and joint stiffness or a history of total hip arthroplasty may have difficulty achieving this position, and the well leg may be at risk of injury in a hemilithotomy position. A previously described position called the “scissor position” is, in our opinion, a safer and more efficient technique for placing the well leg on the orthopedic table, using only a pillow and a self-adhesive compression bandage. This simple position allows a lateral fluoroscopic view of the injured femur without overlapping or interference from the other limb.

## Introduction

Since the new century, orthopedic procedures show a great increase [1]; most of these surgeries are due to hip fractures. This is a major public health problem and is associated with a decrease in quality of life and considerable mortality [2-4]. Chile is no exception, and a recent study has shown that patients older than 50 years have a lower probability of dying one year after hip fracture, even more so if they are treated in a private health center [2]. Trochanteric and subtrochanteric hip fractures are more common in women than in men, and the 1-year mortality rate is as high as 26% [3]. The most common type of fracture, according to the Arbeitsgemeinschaft für Osteosynthesefragen/Orthopaedic Trauma Association (AO/OTA) classification system, is type 31-A2, and the majority of patients undergo intramedullary nailing [3-4]. Percutaneous techniques are the most commonly used techniques, and the quality of reduction remains paramount [5]. Therefore, it is critical that the patient is positioned appropriately for surgery and adequate intraoperative visualization is achieved.

Several surgical positions have been described: supine on a fracture table, supine on a flat radiolucent table, lateral decubitus position on a flat radiolucent table, and the prone position for intramedullary nailing of subtrochanteric femoral fractures [6,7]. The position is selected on the basis of the type of lesion, the classification of the lesion, the degree of involvement of other structures and soft tissues, and finally, the surgeon’s experience and preference. However, most cases of lateral hip fractures are resolved on an orthopedic table [4]. This table allows the degrees of traction and rotation necessary for the adequate reduction of fractures. The use of intraoperative radioscopy in two orthogonal planes is essential to verify that adequate reduction has been achieved and the internal fixation implants have been placed correctly. For an axial view of the hip, it is necessary to find the best projection. Classically, and in most publications, the other limb is positioned on support in hip flexion, hip abduction, hip external rotation, and knee flexion (the hemilithotomy position). However, complications such as compartment syndrome [8-11] and common peroneal nerve palsy [12] of the healthy limb have been described. Furthermore, a survey study showed there is no consensus among major trauma centers in North American countries regarding the optimal patient position for femoral intramedullary nailing [13]. Only 56% of the surveyed orthopedic surgeons preferred the supine position in the fracture table, with the other limb in the hemilithotomy position. However, they reported malreduction in 68% of cases, pudendal nerve injury in 2% of cases, and other complications.

Bible and Mir [14] reported a position that was found to be safer for the contralateral leg and did not hinder the use of radioscopy. They positioned the well leg in slight extension along the traction bar of the table, placed a pillow sling between the bar and the limb, and secured the limb with straps. They used this technique to treat several types of fractures, including femoral neck fractures, intertrochanteric or subtrochanteric fractures, and femoral shaft fractures. They did not report any perioperative complications. Moreover, this position may be advantageous in the management of subtrochanteric fractures, for which a large traction force is required, since the well leg being placed in extension can create countertraction and thus prevent the pelvis from inclining, allowing access to the appropriate proximal entry point [15-16].

In our experience, it is not uncommon to encounter patients who previously underwent operations on the contralateral hip, including total hip arthroplasty or hemiarthroplasty, and patients with hip stiffness secondary to osteoarthritis. For these patients, positioning the hip in flexion and internal rotation for the insertion of the C-arch in the axial view can be risky, and many times, an adequate position is not possible to achieve, resulting in unsatisfactory axial vision and an increased risk of poor reduction.

For this reason, for most cases of hip fractures that we treated over the last year, we preferred to use the scissor position.

## Technical report

The objective of this technical report is to describe the surgical technique and the results of using the scissor position on an orthopedic table for the reduction and internal fixation of intertrochanteric and subtrochanteric hip fractures.

Operative technique

**Figure 1:**
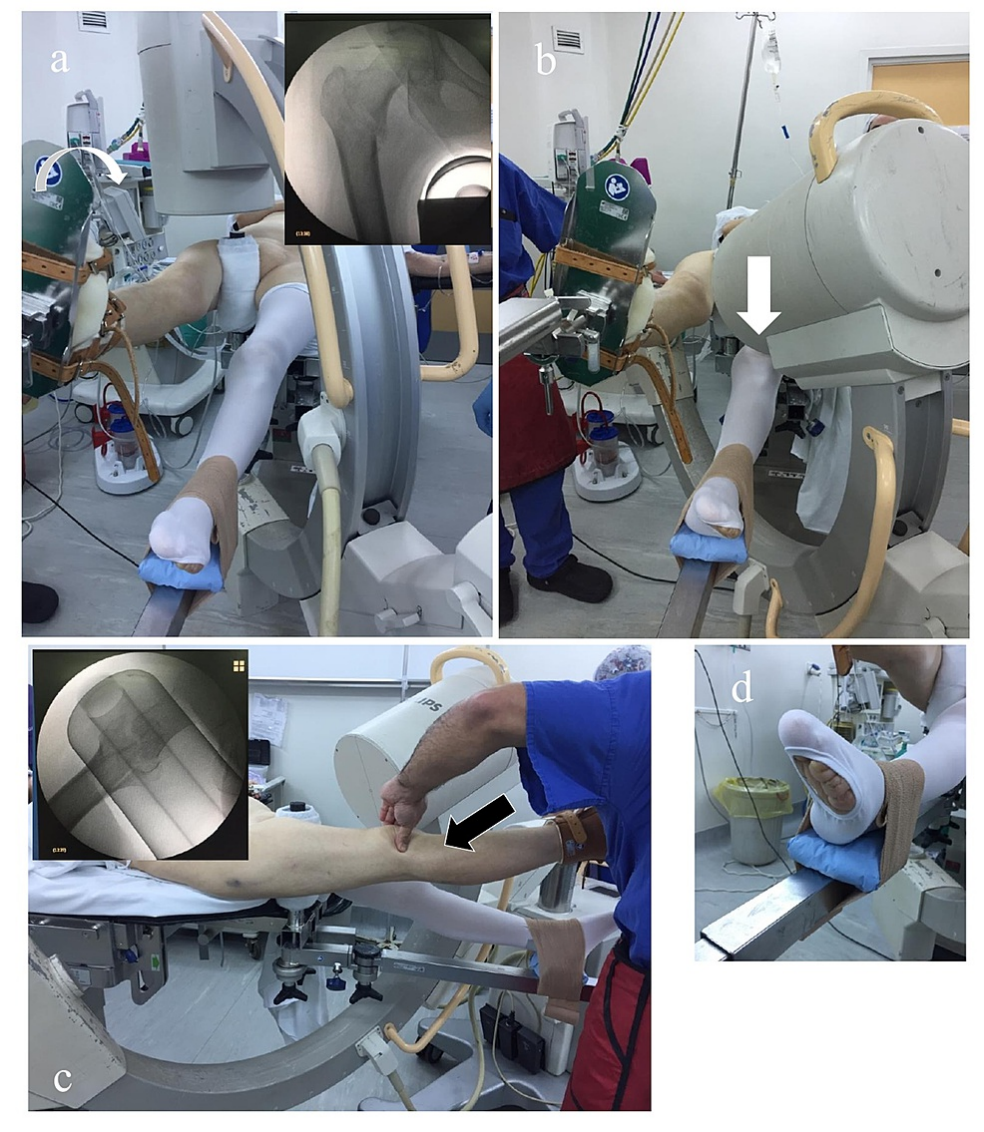
Intraoperative C-arm position Intraoperative C-arm position. (a) While the table is in the scissor position, the C-arm shows the anterior-posterior view. The white arrow shows that internal rotation is required for fracture reduction. The inset picture shows the fluoroscopic anteroposterior (AP) view. (b) The C-arm is positioned at 75 degrees of tilt for the true axial view. The white arrow shows there is adequate space between the well leg and the C-arm. In (c), the position of the C-arm is observed from the side for axial vision. The inset picture shows the true axial fluoroscopic view, with the neck and the femoral shaft being aligned correctly, and the black arrow shows the patella being palpated as a guide for the correct degree of rotation of the limb. In (d), the well-leg foot is placed on the bar and protected by a pad and a bandage.

The patient is placed in the supine position on an orthopedic table. Both feet are draped and protected with cotton or soft bandages. The operated limb is left uncovered, secured in the orthopedic table boot, and prepared for pulling maneuvers. The contralateral or well leg is covered with antithrombotic long-socks and is positioned in slight extension, with the heel on the support bar of the table (Figure 1).

**Figure 2:**
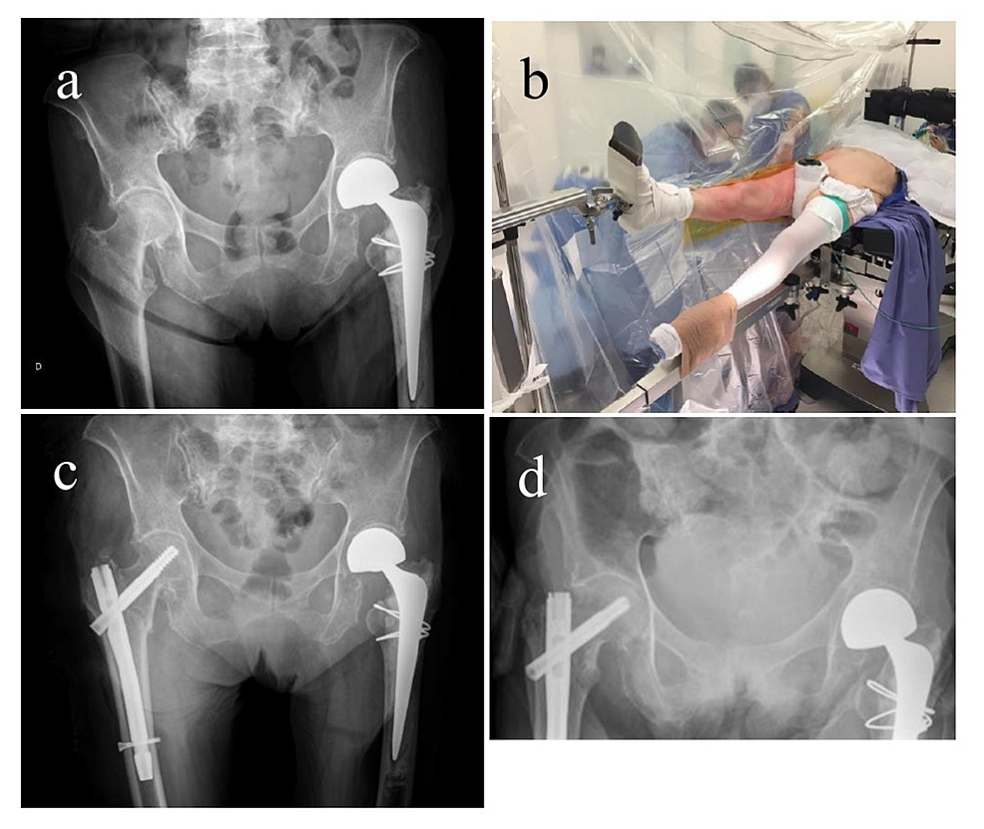
Left hip hemiarthroplasty and right intertrochanteric fracture (a) An 81-year-old female patient; preoperative anteroposterior pelvic radiographs demonstrating a history of left hip hemiarthroplasty and a right intertrochanteric fracture. (b) Example of the scissor position on a fracture table using a small pad and a bandage to protect and secure the foot of the well leg. (c) Immediate postoperative pelvis X-ray and (d) 10-month follow-up X-ray.

The heel is protected with a small pad and secured with a bandage, which is not overtightened to prevent skin damage. The C-arm for radioscopy is then introduced to visualize the anteroposterior and true-axial views. Under these views, the degrees of traction and rotation necessary for the reduction of the fracture is applied without mobilizing the contralateral extremity or obstructing the radioscopic views. We routinely use a transparent vertical field, which allows the operator of the radioscopy equipment to observe both exposed limbs at any given time (Figure 2).

Patients and Methods

We have used this position since January 2019 for most lateral hip fractures, especially in patients in whom we suspected joint stiffness or who had an implant and we wanted to avoid flexion and external rotation.

We present a retrospective clinical study of 118 patients with intertrochanteric and subtrochanteric hip fractures who underwent surgery in this “scissor position” on an orthopedic table. Our inclusion criteria consisted of patients over 18 years and no contraindication for surgery.

The Hospital San José Ethical Review Authority approved the study and appropriate written consent was obtained from all patients. Our study was performed in accordance with relevant named guidelines and regulations.

 

Results

**Figure 3:**
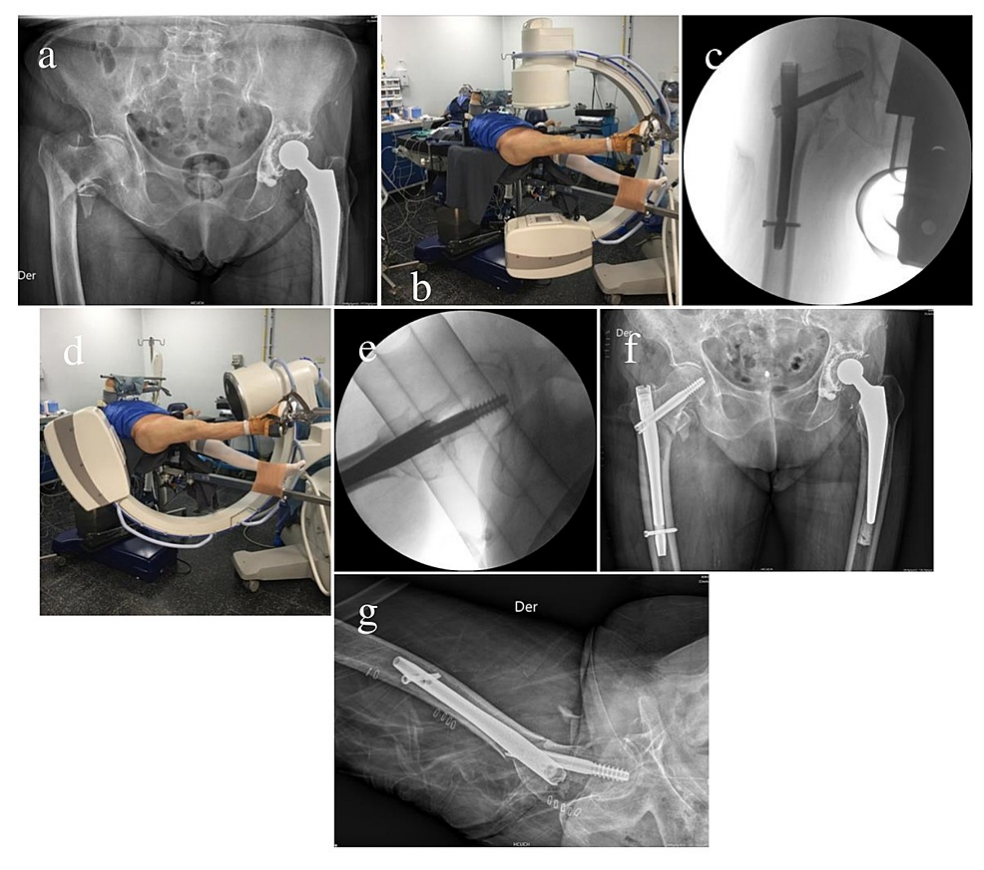
Left total hip arthroplasty and right intertrochanteric fracture (a) For an 85-year-old female patient, the anteroposterior (AP) radiograph shows that left total hip arthroplasty had previously been performed and there was a right intertrochanteric fracture. (b) The well-leg scissor position on the fracture table and C-arm in the AP position. (c) AP fluoroscope of the cephalous nail system after fracture reduction. (d) Well-leg scissor position and C-arm in the axial view. (e) Axial fluoroscope view of the cephalous nail system after fracture reduction. (f and g) Immediate postoperative pelvis X-ray in the AP and axial views.

**Figure 4:**
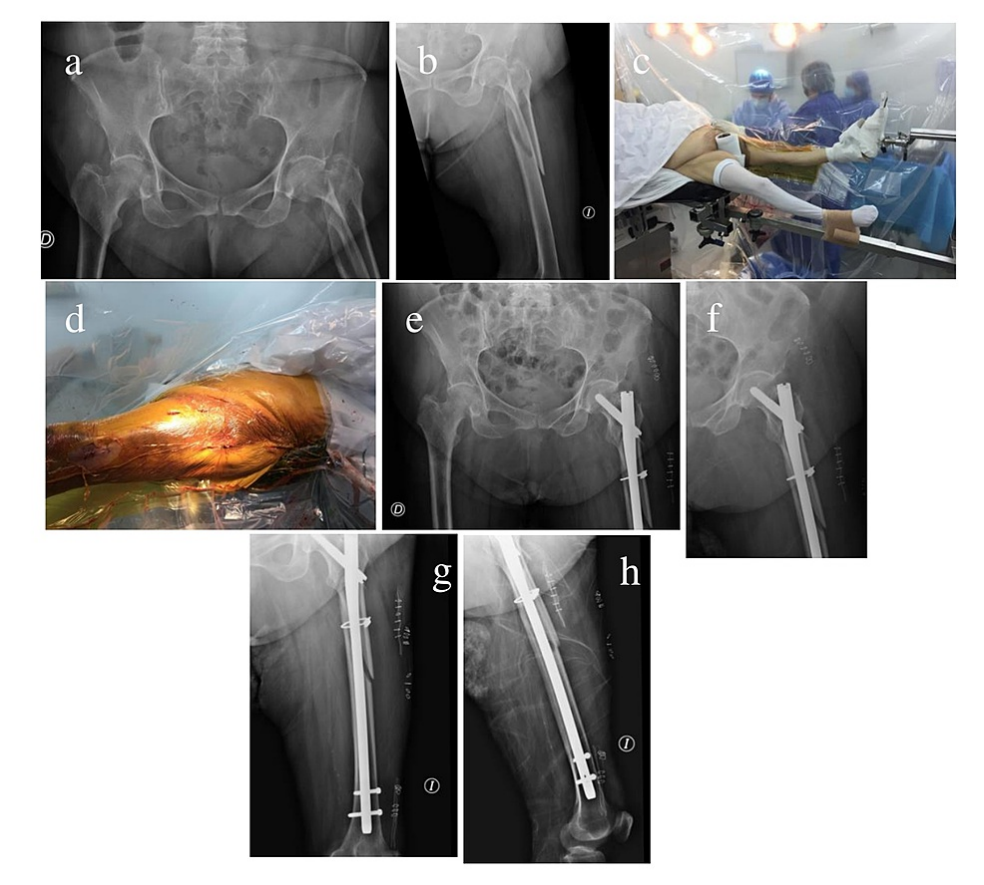
Left subtrochanteric fracture and well leg scissor position (a and b) A 75-year-old female patient; preoperative anteroposterior (AP) pelvis and femur X-rays demonstrating a left subtrochanteric fracture. (c) Well leg scissor position and a transparent vertical field. (d) Transparent vertical field on the surgeon’s side showing mini incisions in the operated femur. (e-h) Immediate postoperative pelvis and femur X-rays in the AP and lateral views.

The mean age of the 118 patients was 79 years (33-103 years), and 86 were women. A total of 112 patients had intertrochanteric fractures, and 6 had subtrochanteric fractures. The fractures were classified according to the OTA system, and there were 104 type A2 intertrochanteric fractures, for which a short spinal cephalous nail system (Gamma, Stryker) was used. A dynamic hip screw (DHS) (DePuy, Synthes) was used for the 8 type A1 fractures with an adequate lateral wall. A long nail (Gamma, Stryker) was used for all subtrochanteric cases. The average operating time was 38 minutes (15-175 minutes). We did not observe any intraoperative complications, and there was no discomfort or cases of compartment syndrome, nerve paralysis, or clinical deep vein thrombosis in the contralateral limb. The radiological results were satisfactory in most of the cases (83%) (Figure 3 and 4).

## Discussion

Patients must be placed in safe and appropriate operative positions for the resolution of hip fractures [4-5]. In addition, good radiological views are crucial for the success of current percutaneous fixation techniques. Patients who previously underwent surgeries or present with difficulties may require a position different from the position that we usually use to obtain satisfactory fluoroscopic views [11-12]. In this article, we present technical advice for the surgical resolution of inter and subtrochanteric fractures, using the “scissor position” on a traction-orthopedic table. In particular, in cases with hip stiffness or a history of contralateral surgery, the hemilithotomy position can be difficult and hinder adequate C-arm views.

The hemilithotomy position, also called the Lloyd-Davis position, involves hip flexion, hip abduction, hip external rotation, and knee flexion. It is the most commonly used supine position for treating hip fractures. However, there are several reports of complications associated with the hemilithotomy position. Brouze et al. [8] described two cases of well leg compartment syndrome associated with the hemilithotomy position in young patients during the femoral nailing of femur diaphyseal fractures. This complication is rare and associated with surgical time, body mass index, and the hemilithotomy position. Both patients underwent emergent fasciotomy of the four compartments in the leg. The authors suggest that every patient whose surgery lasts more than 2 hours is considered at risk of developing this complication, and they recommend measures such as reducing the degrees of flexion of the hip and knee, mobilizing the uninjured leg every 2 hours, avoiding firm support at the level of the knee or calf, keeping the ankle free and not prescribing hypovolemia and vasoconstrictor agents. Furthermore, Hsu et al. [12] reported an unusual case of contralateral common peroneal nerve palsy in a 28-year-old female who developed drop foot after femoral nailing. The authors attributed this case to a possible iatrogenic neuropathy secondary to the supine hemilithotomy position and compression in the immobilization boot, with the knee flexed by more than 90°.

There are several publications that can help surgeons correctly perform nailing for intertrochanteric, subtrochanteric and femur fractures. Firat et al. [17] developed a technique of positioning the patient supine with the contralateral leg elevated. Although this method led to a short operative duration, it can still be problematic for our older patients with fractures because of hip stiffness and an increased rate of hip arthroplasty having been performed in the contralateral limb. Kasha and Yalamanchili [15] reviewed several nailing techniques for subtrochanteric fractures and recommended avoiding the hemilithotomy position to prevent malalignment of the injured proximal femur. The countertraction of the well leg allows better access to the entry point of the nail. In addition, in cases of severe subtrochanteric comminution, the contralateral lower limb can be used as a reference for hip rotation, the patella position, and the ankle position. On the other hand, Sharma et al. [18] reported a technique of positioning the well leg over the C-arm for distal locking during femoral nailing. They started with a Figure 4 position with the uninjured hip flexed to 90 degrees, abducted, externally rotated, and rested in a well-padded gutter splint to allow full access to the proximal femur. Then, for distal locking, the authors stated that positioning the uninjured leg over the C-arm is an alternative option while distal locking for femoral nailing is performed. The advantages include the method being safer for hips that have previously been operated on and an inherently lower risk of dislocation compared with the position of the uninjured leg in the previously mentioned two positions [15-18]. However, in our opinion, it would be ideal to not position the well leg in hip flexion, abduction, or external rotation, so that patients who previously underwent arthroplasty are not at an increased risk of dislocation. The scissor position allows adequate C-arm mobility, good access for axial views, and a safe position for proximal locking.

The mean operating time in our study was 38 minutes, which is in concordance with that in the literature. In a study of 27 patients, Brin et al. [19] reported a reduction in radiation exposure by 29% (51.2 ± 18.9 s to 36.6 ± 8.6 s) and a reduction in the total operation time by 30% when they used two fluoroscopes (axial and anteroposterior (AP)) at the same time rather than one; we conventionally use only one fluoroscope. Additionally, Mastrangelo et al. [20] stated that there is an increased risk of orthopedic surgeons developing cancer, especially due to exposure to radiation. The recommend radiation time exposure is decreased; we used a C-arm with high maneuver capability and a wide field of vision, which yields satisfactory results in 83% of cases.

The limitations of this report are the retrospective design of the study and the lack of a control group. Comparative studies are needed to demonstrate whether this position is superior to the others. However, in our opinion, this position is an excellent alternative for the intraoperative fluoroscopic view, and we did not observe any associated complications.

## Conclusions

The scissor position is a safe and efficient technique; the well leg is positioned on the table using a pillow and a self-adherent compression bandage. This position prevents potentially devastating complications and enables lateral fluoroscopic views without overlapping of the well leg or interference with the C-arm.
